# Genetic improvement of non-conventional *Torulaspora delbrueckii* for traditional sparkling winemaking by mixing for eventual hybridization with *Saccharomyces cerevisiae*

**DOI:** 10.3389/fmicb.2022.1006978

**Published:** 2022-10-06

**Authors:** Alberto Martínez, Emiliano Zamora, María L. Álvarez, Joaquín Bautista-Gallego, Manuel Ramírez

**Affiliations:** ^1^Departamento de Ciencias Biomédicas (Área de Microbiología), Facultad de Ciencias, Universidad de Extremadura, Badajoz, Spain; ^2^Estación Enológica, Junta de Extremadura, Almendralejo, Spain

**Keywords:** *Torulaspora delbrueckii*, *Saccharomyces cerevisiae*, wine fermentation, sparkling wine, ethanol resistance, SO_2_ resistance, pressure resistance

## Abstract

Non-conventional yeasts such as *Torulaspora delbrueckii* (*Td*) have been proposed for sparkling winemaking. Unfortunately, this yeast has poor efficiency in completing wine fermentation as compared to *Saccharomyces cerevisiae* (*Sc*). New mutants with increased resistance to SO_2_, ethanol, and high CO_2_ pressure were previously isolated from spore clones of *Td*. Although these mutants showed improved capability for base wine fermentation, there is still room for genetic improvement of *Td* yeasts until the fermentative capacity of *Sc* is achieved. As an alternative approach, yeast mixture for eventual hybridization of *Td* with *Sc* was assayed in this study. The new yeast mixture clones (*Sc*-mixed *Td*) showed an intermediate phenotype between both parent yeasts for some relevant biotechnological properties, such as resistance to SO_2_, ethanol, copper, high CO_2_ pressure, and high temperature, as well as flocculation potential. These properties varied depending on the specific *Sc*-mixed *Td* clone. Several mixture clones showed improved capability for base wine fermentation as compared to the *Td* parent strain, approaching the fermentation capability of the *Sc* parent strain. The organoleptic quality of sparkling wine was also improved by using some mixture clones and this improved quality coincided with an increased amount of acetate and ethyl esters. The genetic stability of some *Sc*-mixed *Td* clones was good enough for commercial yeast production and winery applications.

## Introduction

*Torulaspora delbrueckii* (*Td*), a non-conventional yeast that is being recommended for must fermentation, can change the amount of some compounds to improve the quality and complexity of wine (Benito, 2018; Ramírez and Velázquez, 2018; Morata et al., 2022). This yeast can decrease volatile acidity and ethanol production, increase the amount of glycerol, increase mannoprotein and polysaccharide release, increase the amount of interesting aromatic compounds (such as fruity esters, lactones, thiols, and terpenes), and decrease the amounts of some undesired compounds such as higher alcohols. It can also promote malolactic fermentation (Ramírez et al., 2016; Balmaseda et al., 2018). Despite this, *Td* strains have some disadvantages for winemaking because it has greater rates of CO_2_ production and O_2_ consumption than the conventional wine yeast *Saccharomyces cerevisiae* (*Sc*). This decreases the biomass yield from industrial yeast culturing, which is a concern for *Td* commercial production (Mauricio et al., 1998). As *Td* grows less or slower than *Sc* under anaerobic conditions (Visser et al., 1990; Hanl et al., 2005), it shows less fermentation vigor than *Sc* during wine fermentation, and it has difficulty to dominate must fermentation even when inoculated at high amount, above 10^7^ CFU/ml (Mauricio et al., 1998; González-Royo et al., 2015; Ramírez and Velázquez, 2018). These drawbacks are specifically relevant for sparkling winemaking, which is performed under strict anaerobic conditions (Ramírez and Velázquez, 2018). Furthermore, *Td* is poorly resistant to other stressful conditions associated with sparkling winemaking, such as high ethanol concentration, presence of SO_2_, and increasing pressure of CO_2_. Therefore, the efficiency of *Td* for sparkling wine fermentation is rather low, mostly because cell death increases quickly after base wine inoculation (García et al., 2016; Velázquez et al., 2019). Consequently, wine fermentations single inoculated with *Td* tend to slow to become sluggish, stop, or eventually continue due to the involvement of some contaminating *Sc* yeasts (González-Royo et al., 2015; Velázquez et al., 2015). This reduced participation of *Td* during base wine fermentation makes uncertain the actual effect of this yeast on sparkling wine quality (Benito, 2018; Velázquez et al., 2019).

Among all those stressful conditions, SO_2_ is generally used in winemaking as antioxidant and antimicrobial agent (García-Ríos and Guillamón, 2019). Therefore, SO_2_ resistance is a desired trait for sparkling wine yeasts. Usually, *Td* resists lower SO*_2_* concentration than *Sc*. Although *Td* may survive and complete must fermentation in the presence of 50 mg/l SO_2_, it uses to die in the presence of 125 mg/l SO_2_ (Ramírez and Velázquez, 2018). As base wine usually contains SO_2_, this is an issue to consider for preserving *Td* cell viability during base-wine second fermentation.

Attempts to use several *Td* strains for sparkling winemaking revealed that they are unable to complete base-wine second fermentation. They did not survive CO_2_ pressure above 3.5 atm inside the glass bottle (Velázquez et al., 2019). Base wine single-inoculated with *Td* only completed the second fermentation when contaminating *Saccharomyces* yeasts were involved in the process (Ramírez and Velázquez, 2018). Therefore, the resistance of *Td* to high CO_2_ pressure should be increased to improve the dominance of this yeast during sparkling wine second fermentation.

An attempt to solve the above-mentioned drawbacks of *Td* was done by elimination of possible recessive deleterious alleles to get improved *Td* spore-clones with enhanced fermentation capability. Additionally, sequential isolation of spontaneous mutants resistant to the mentioned stressful conditions was thereafter performed to bring the overall fermentation performance of *Td* as close as possible to that of *Sc* wine strains. New mutants resistant to SO_2_ and ethanol with slightly improved fermentative efficiency in base wine were obtained (Velázquez et al., 2020). However, a new isolation of HP^R^ (high pressure resistant) mutants from these mutants already resistant to SO_2_ and ethanol was required to get new *Td* strains with relevant improved efficiency for sparkling wine second fermentation (Velázquez et al., 2020). Despite this interesting advance in improving the fermentative capacity of *Td* by obtaining HP^R^ mutants, there is still room to improve the fermentative capacity of *Td* yeasts to reach that of *Sc* wine yeasts. As an alternative strategy, one can think of the possibility of hybridizing *Td* with *Sc* to improve the biotechnological properties of the former.

Hybridization of diploid strains is a suitable method to consider for improvement of yeasts included in the *Saccharomyces sensu stricto* taxon. These yeasts have similar genomes (32 chromosomes in diploid phase) and close phylogenetic relationship. For heterothallic strains, it is easy to obtain hybrids by micromanipulating the zygotes formed between meiotic segregants with complementary mating types. For homothallic diploid yeasts, as *Sc* wine strains (Romano et al., 1985; Bakalinsky and Snow, 1990; Mortimer et al., 1994; Guijo et al., 1997; Ramírez et al., 1999), hybridization can be accomplished by mixing sporulated cultures (Romano et al., 1985). Yeast mating may occur between spore germination and diploidization. However, hybrids are obtained with low frequency, and they are difficult to identify. To facilitate hybrid selection and identification, one can take advantage of the killer phenotype that is frequent among wine yeasts (Shimizu, 1993; Vagnoli et al., 1993; Hidalgo and Flores, 1994; Da Silva, 1996; Ramírez et al., 2015, 2017, 2020, 2022). It is possible to change the culture conditions to make yeasts conjugate or kill each other, so that many hybrids can easily be obtained (Ramírez et al., 1998). Among non-conventional yeasts as *Td*, the lack of genetic knowledge is detrimental at the time to face a genetic improvement by obtaining hybrid yeasts sharing biotechnological properties from two different parent strains. Recent advances in high-throughput sequencing (HTS) have developed new genomic and genetic tools for non-conventional yeasts, but these are not so efficient as those traditionally used for the conventional *Sc* yeast (Masneuf-Pomarede et al., 2016). The main handicap is the lack of precise knowledge about the life cycle of non-conventional yeast such as *Td* to design strategies for biotechnological improvement by using the classical genetic techniques already used for *Sc* wine strains (Ramírez et al., 1998, 1999; Ramírez and Rebollo, 2003; Ramírez and Ambrona, 2008). Hybridization between *Td* and *Sc* wine yeasts could be assayed to get some interesting properties of *Sc* to be transferred to *Td*. If this is pursued, one should take into account that *Td* wine strains are haploid yeast containing half of the chromosome present in haploid cells of *Sc* (8 vs. 16). Therefore, the obtaining of true genetically stable hybrids is not likely at all, but the transfer of compatible genetic information from *Sc* to *Td* could be expected. However, the loss of interesting traits from *Td* during the hybridization process could also be possible.

In this work, several mixing for eventual hybridization of spores and vegetative cells of a *Td* killer strain (Kbarr-1) with a *Sc* cycloheximide resistant (CYH^R^) strain were done. Characterization of yeast mixture clones was performed to ensure the transfer of genetic information from *Sc* to *Td*. The fermentation capability of some selected mixture clones was analyzed for comparison with that of the parent yeasts. The main aim was to improve the base wine fermentation performance of *Td* to bring it as close as possible to that usually shown by the *Sc* strains used for sparkling winemaking. The feasibility of some *Sc*-mixed *Td* clones for commercial winery applications is addressed.

## Materials and methods

### Yeast strains

*Saccharomyces cerevisiae* (*Sc*) 85R4A is a spore-clone, non-killer, cycloheximide-resistant (CYH^R^), prototrophic wine yeast obtained from JP85R. This strain was used in this study as parent for yeast mating and as reference yeast for sparkling wine fermentation. *T. delbrueckii* (*Td*) EX1180 is a killer Kbarr-1 prototrophic wine yeasts that kill all known types of *S. cerevisiae* killer and non-killer strains. These two yeasts were previously selected for winemaking (Velázquez et al., 2015, 2019; Ramírez et al., 2016). The genetic marker CYH^R^ allows making traceability of *Sc* EX85R4A during wine fermentation, as well as the identification of presumptive hybrid yeasts obtained by crossing this yeast with a cycloheximide-sensitive (CYH^S^) yeast. Commercial use of *Td* EX1180 is under patent application (Ramírez et al., 2015).

### Culture media and phenotype tests

Yeast growth was done in standard culture media (Guthrie and Fink, 1991). YEPD-CYH was YEPD-agar supplemented with 2 μg/ml cycloheximide (Pérez et al., 2000). YEPD-EtOH was YEPD-agar supplemented with ethanol before pouring the medium into plates to 10% (*v*/*v*) final concentration. SD-agar contained 0.67% Yeast Nitrogen Base (without amino acids; with ammonium sulfate, Difco), 2% glucose, and 2% Bacto-agar (*w*/*v*). SD + SO_2_ is SD-agar buffered with 75 mM tartaric acid at pH 3.5 and supplemented with a just made 6% K_2_S_2_O_2_ solution 2 h before yeast seeding (125 or 250 mg/l SO_2_ final concentration; Ramírez et al., 1999).

Standard procedures were used for sporulation of yeast cultures (Kaiser et al., 1994). Yeast cells were grown in YEPD broth (1% yeast extract, 2% peptone, and 2% glucose) or on YEPD-agar plate (YEPD broth with 2% agar, *w*/*v*) for 2 days at 30°C and then transferred to sporulation medium (1% potassium acetate, 0.1% yeast extract, 0.05% glucose, *w*/*v*) and incubated at 25°C. Parent and yeast mixture clones were tested for copper resistance, SO_2_ resistance, and H_2_S production as previously described (Mortimer et al., 1994). Killer activity was tested on low-pH (pH 4) methylene blue plates (4 MB; Kaiser et al., 1994) seeded with 100 μl of a 48-h grown culture of a sensitive strain (Ramírez et al., 2004). Alcian Blue test was performed as previously described (Fukudome et al., 2002; Corbacho et al., 2005). Briefly, yeast cells were grown in YEPD broth in a microtiter plate for 24–48 h at 4°C. Subsequently, the microplate was centrifuged at 3,500 rpm for 3 min and each well was washed with 0.9% NaCl (*w*/*v*). Alcian Blue (1% Alcian Blue in 3% acetic acid pH 2.5 from Sigma-Aldrich, diluted 1/10 with 0.02 N HCl) was added, mixed with the cells, and kept at room temperature for 15 min. Yeasts were washed twice with 0.02 N HCl and the plate was scanned to quantify the degree of blue color. This test reflects the presence (blue stain) or absence (no stain) of phosphate in the cell wall mannoproteins.

### Synthetic must and synthetic base wine fermentations

The sterile synthetic must was a modified version (OIV, 2012) of the previously described (Henschek et al., 1993) in such a way that the amount of amino acids was decreased to give a nitrogen concentration of 200 mg/l, the amount of sugar was increased to 230 g/l (instead of 200 g/l), and the amount of vitamins was reduced to one-fifth. This medium was sterile filtered. Sterile synthetic base wine contained 1% yeast extract, 0.1% peptone, 2.4% sucrose, 0.3% tartaric acid, 0.2% malic acid (*w*/*v*), and 10% ethanol (*v*/*v*), pH 3.1. Yeast cells were cultured in YEPD broth for 2 days at 30°C, washed twice with sterile water, and inoculated into synthetic must or synthetic base wine (1–2 × 10^6^ cells/ml for *Sc* 85R4A, and 2–4 × 10^6^ cells/ml) for *Td* yeasts (EX1180, *Sc*-mixed *Td* clones, and *Td* MutHP41). The number of inoculated *Td* cells doubled compared to *Sc* because *Td* shows less fermentation vigor than *Sc* during wine fermentation, as mentioned above. Fermentations of synthetic must and synthetic base wine were performed in 250-ml Erlenmeyer flasks with 60 ml of base wine, at 20°C or 18°C, respectively. Before base wine inoculation, yeast cultures were adapted to grow in this medium as indicated below for sparkling winemaking. Where indicated, continuous instead of occasional shaking was used to increase the availability of oxygen (Velázquez et al., 2020). °Brix, yeast growth (total and viable yeast cells), and dead cells were monitored. The amount of glucose + fructose was measured at least at the end of each experiment to check for the completion of fermentation. Cell death was determined by staining with methylene blue and observation of yeast cells under a microscope. Since the morphological changes in the yeast cells during the second fermentation of base wine used to be very variable, the total amount of dead cells was calculated as the sum of blue, empty, and destroyed/autolyzed cells (Velázquez et al., 2019).

### Sparkling winemaking with commercial base-wine

Cava-type sparkling wine was produced by the traditional method in collaborating wineries and our experimental winery as previously described (Velázquez et al., 2019). Three different commercial base wines were used, all of them from *Macabeo* white grapes. Macabeo I (pH 3.18, 5.7 g/l total acidity, 0.83 g/l reducing sugars, 10.8% alcohol *v*/*v*, 90 mg/l SO_2_) and Macabeo III (pH 3.17, 5.5 g/l total acidity, 1 g/l reducing sugars, 9.4% alcohol *v*/*v*, 61 mg/l SO_2_) base wines were from Bodegas López Morenas (Almendralejo, Spain). Macabeo II base wine (pH 3.16, 5.3 g/l total acidity, 0.9 g/l reducing sugars, 10.2% alcohol *v*/*v*, 72 mg/l SO_2_) was from Bodegas Romale (Almendralejo, Spain). These base wines were freshly made during the previous grape harvest (2018 for Macabeo I and II, and 2020 for Macabeo III), as it is usual for cava sparkling wines. As consequence, they contained about 1 × 10^2^ CFU/ml contaminant *S. cerevisiae*-like yeasts. Prior to base wine inoculation, each yeast culture was adapted to growth in base wine as previously described (Velázquez et al., 2020). Agitation of the yeast cultures was eventually done every 2–12 h during the adaptation process. The adapted yeast cultures contained 2–6 × 10^8^ CFU/ml. For sparkling winemaking, commercial base wine was supplemented with 2.4% sucrose and 0.02% diammonium phosphate, and single-inoculated with each adapted yeast culture in 0.75 l capped bottles, in which high pressure above 6 atm could be reached after the second fermentation. At least 50 replicates of each yeast second fermentation were done. The intended amount of yeast inoculum was 1–2 × 10^6^ viable cells/mL for *Sc*, 2–4 × 10^6^ viable cells/ml for *Td*, and 1–2 × 10^6^ viable cells/ml of *Sc* plus 2–4 × 10^6^ viable cells/ml of *Td* for co-inoculation with *Sc* 85R4A + *Td* EX1180 (1,2). Second fermentation was done at 18–19°C for the first 15 days, and thereafter at 12–14°C to preserve the wine organoleptic quality. Samples for microbiological and chemical assays were taken at different times from 0 to 270 days. After 270 days of fermentation and aging, sparkling wines were riddled for 40 days to move the lees to the bottleneck. Finally, after disgorging, aromatic compounds and organoleptic quality assays were done. A global organoleptic evaluation was done for each sparkling wine by an expert panel of ten judges as previously described (Velázquez et al., 2016). The judges scored the quality of the wines on a six-point scale (0 = very poor, 1 = deficient, 2 = acceptable, 3 = good, 4 = very good, and 5 = excellent). The maximum score possible (50 points) was considered 100% preference. All wine evaluations were done in duplicate.

### Determination of the proportion of inoculated yeast during wine fermentation

The percentage of genetically marked CYH^R^ yeasts as *Sc* 85R4A and *Sc*-mixed *Td* clones was determined by replica-plating on YEPD + CYH plates (Velázquez et al., 2016). Additionally, resistance to 40°C, spore morphology, killer phenotype, and H_2_S production were also used to validate the results from YEPD+CYH replica-plating. The percentage of wild yeasts as *Td* EX1180 was determined by mtDNA restriction pattern analysis (Maqueda et al., 2010). Eventually, this same procedure was also used to validate the results obtained from YEPD+CYH replica-plating of *Sc* 85R4A and *Sc*-mixed *Td* clones.

### Analytical methods

Brix was measured using a digital refractometer. Alcohol content, pH, total acidity, volatile acidity, glucose + fructose, and density were determined using EC recommended methods (EC, 1999). Sparkling wine pressure was measured at room temperature using an aphrometer, and values were then corrected to 20°C by using the Henry’s law constant. Wine aroma compounds were isolated and pre-concentrated by a solid-phase extraction (García-Carpintero et al., 2011), and then assayed by gas chromatography–mass spectrometry as previously described (Velázquez et al., 2015). Quantitative data was obtained by calculating the peak area of each compound compared to that of the internal standard, interpolating with the corresponding calibration plot that was constructed from analysis of known amounts of volatile aroma standards. For compounds whose authenticated standards were not available (ethyl 9-decenoate, diethyl 2-hydroxyglutarate, ethyl 2-hydroxy-3-phenylpropanoate, and γ-ethoxy-butyrolactone), identification was based on spectral comparison with the Wiley A library data, and quantification was performed using the calibration curves of standards with similar chemical structures obtained in the TIC mode. The identity of the 75 detected compounds and the odor activity value (OAV) calculation were described previously (Velázquez et al., 2015).

### Miscellaneous

DNA manipulations (enzyme digestions, PCR, and gel electrophoresis) were done in accordance with standard methods (Sambrook et al., 1989). Sequencing of ITS (internal transcribed spacer region of the nuclear ribosomal repeat) DNA was done at the STAB (Servicio de Técnicas Aplicadas a la Biociencia) at the University of Extremadura (Badajoz, Spain) as previously described (Toju et al., 2012; Gonçalves Dos Santos et al., 2017). Briefly, primers ITS1 (5′-TCCGTAGTGAACCTGCGG-3′), ITS2 (5′-GCTGCGTTCTTCATCGATGC-3′), ITS3 (5′-GCATCGATGAAGAACGCAGC-3′), and ITS4 (5′-TCCTCCGCTTATTGATATGC-3′) were used to amplify the partial nrRNA gene that includes the internal transcribed spacers (ITS 1 and ITS 2) domains, and the intervening region of 5.8S rRNA domain. The PCR products were purified with ExoSAP-IT^™^ PCR Product Cleanup Reagent (Thermo Fisher Scientific, Waltham, MA, United States) and sequenced. The rDNA gene sequences were analyzed against those in GenBank using the BLAST algorithm. Sequences of > 99% similarity with data available at NCBI^1^ were considered to come from the same species. Most enzymes were of the Promega and Sigma brands. Synthetic oligonucleotides were from Biomers. Cell cycle analysis of yeasts was done as previously described (Fortuna et al., 2001) by the Facility of STAB at the University of Extremadura (Badajoz, Spain). Briefly, yeasts were fixed in cold 70% ethanol for 2 h. After washing in PBS, yeasts were resuspended in 1 ml of staining buffer (1/10,000 SYBR Green from Life Technologies, 0.1% RNAse from Sigma, in PBS) and incubated overnight at 37°C. Finally, cells were analyzed in a MACSQuant VYB flow cytometer (Miltenyi Biotec) at no more than 200 events per second, and 200,000 events were acquired per sample.

Data were analyzed for statistical significance by a one-way analysis of variance (ANOVA; *p* < 0.05) with the software package SPSS version 20.0 for Windows (Chicago, IL).

## Results

### Obtaining and characterization of new *Sc*-mixed *Td* yeasts

**Figure 1:**
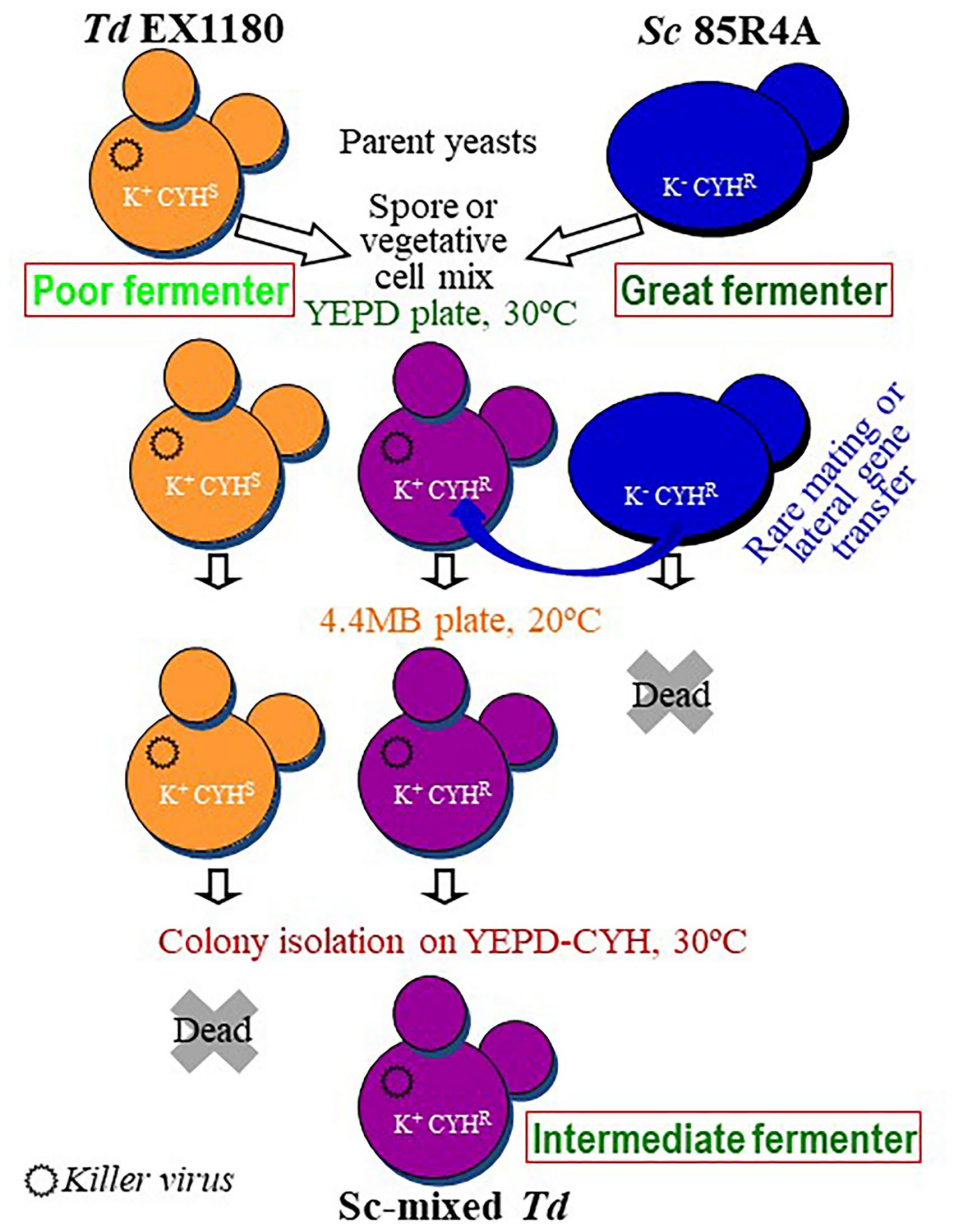
Scheme of the procedure for wine yeast mixture for eventual hybridization. The cell types present in each step are illustrated as schematic yeasts with the relevant phenotypes inscribed., TdV-Mbarr-1 killer virus.

Sporulated and vegetative liquid cultures of *Td* EX1180 (killer Kbarr-1, CYH^S^) and *Sc* 85R4A (sensitive to Kbarr-1 toxin, CYH^R^) were mixed (1,1), plated on YEPD, and incubated at 30°C to allow yeast mating for 2 days. Under these conditions, Kbarr-1 killer toxin is inactive, *Td* killer cells do not kill the *Sc*-sensitive cells, and they can mate with each other. Thereafter, the mixed cultures from the YEPD plates were inoculated onto 4 MB plates and incubated at 20°C for 4–5 days. Under these conditions, the Kbarr-1 toxin is active and kills the parent-sensitive *Sc* 85R4A CYH^R^ cells. However, this toxin does not kill the *Td* EX1180 Kbarr-1 CYH^S^ parent cells, nor the Kbarr-1 CYH^R^ yeasts that may have arisen from yeast mating or lateral gene transfer from *Sc* 85R4A to *Td* EX1180. Single-cell colonies were isolated from these mixed cultures (after growing on 4 MB plates) by spreading some samples onto YEPD and YEPD-CYH plates followed by incubation at 30°C. The isolated colonies were replica-plated on YEPD-CYH followed by incubation at 30°C (Figure 1). As expected, all colonies isolated in YEPD plates from the control CYH^R^ parent strain (*Sc* 85R4A) grew in YEPD-CYH. In contrast, no colonies from the Kbarr-1 CYH^S^ parent strain (*Td* EX1180) appeared on YEPD-CYH plates, and no isolated colonies on YEPD plates from this yeast grew after replica-plating on YEPD-CYH plates. The results from the mixed cultures of both parent strains were variable. No CYH^R^ colonies were obtained from the mixtures of sporulated cultures. However, isolated CYH^R^ colonies arose after 2–3 days on YEPD-CYH from mixtures of vegetative cells of both parent yeasts, with an average frequency of 10^−5^ of the tested yeast cells. The killer phenotype of 20 CYH^R^ isolated colonies was analyzed. All these colonies were killer Kbarr-1, as the colonies from the Kbarr-1 CYH^S^ parent yeast *Td* EX1180. Cell and spore morphology of these yeasts resemble that of the *Td* EX1180 parent yeast (Supplementary Figure S1), and the sequence of the ribosomal RNA gene spacers ITS1 and ITS2 confirmed that they were *Td* yeasts (sequences of > 99% similarity with previously published data available at NCBI,^2^ were considered as the same species). Flow cytometry analysis revealed that they were haploid strains as the parent *Td* EX1180. These yeasts could be haploid segregant yeasts from genetically unstable hybrids raised by any type of rare mating between *Sc* and *Td* vegetative cells. They were named Sc-mixed *Td* yeasts (Sc × Td). Therefore, these yeast mixture clones could be selected *Td* mutants that arose by recombining DNA from the parent yeast *Td* genome with some fragments of the *Sc* genome, at some specific locations of the *Td* genome.

**Figure 2:**
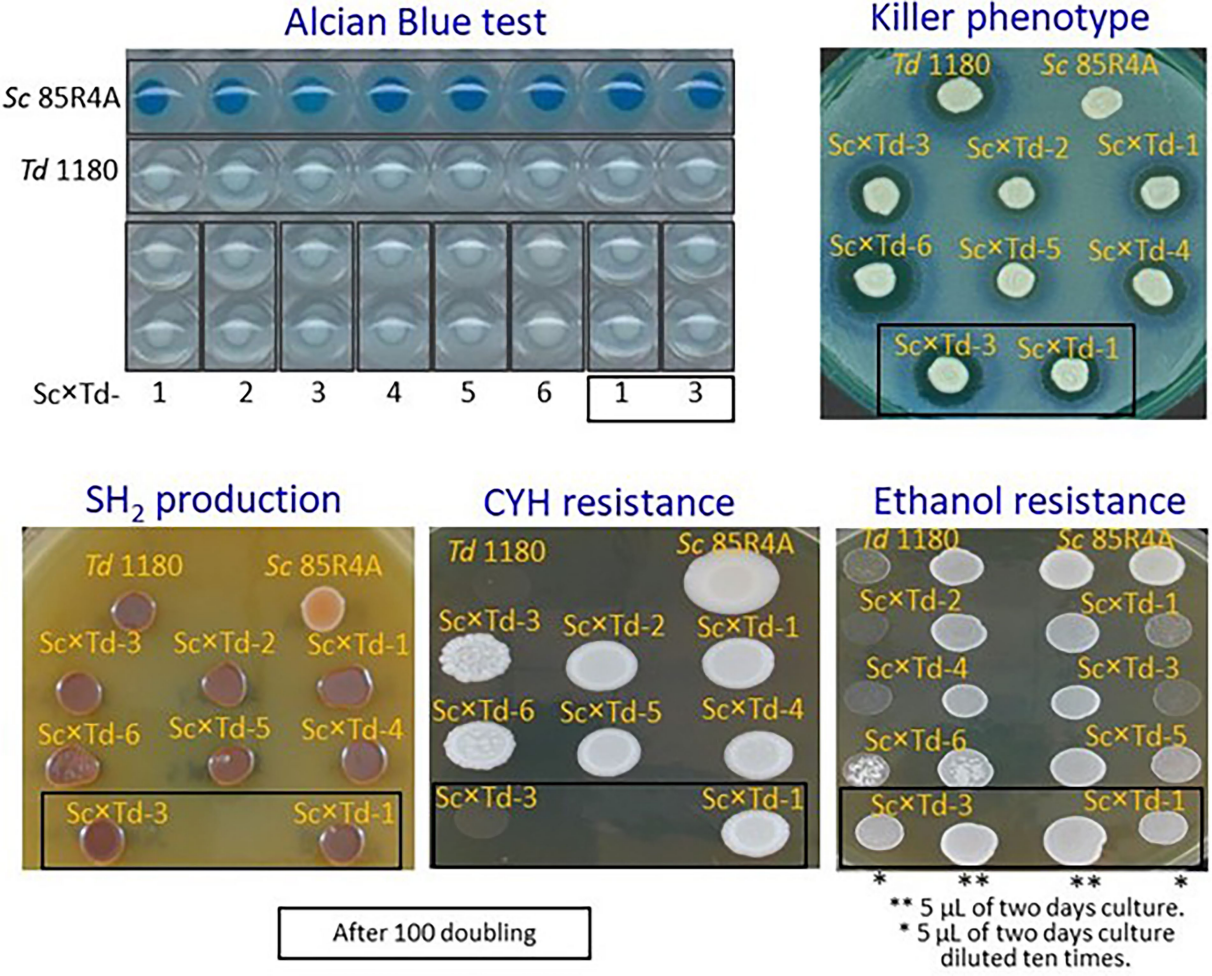
Phenotypic characterization of the *Sc*-mixed *Td* clones and their parent yeasts *Sc* 85R4A and *Td* EX1180. Alcian Blue test reflects the presence (blue stain in *Sc*) or absence (no stain in *Td*) of phosphate in the cell wall mannoproteins. The killer phenotype assay was done on methylene blue agar plates seeded with standard killer-sensitive EX33 yeast. The assay conditions were pH 4, 20°C. H_2_S production was determined on BIGGY agar plate (Oxoid, Basingstoke, Hants, United Kingdom) at 30°C. Dark brown stain means high H_2_S production and light brown or cream color means low H_2_S production. Cycloheximide resistance was tested on YEPD-CYH with 2 μg/ml cycloheximide, incubated at 30°C. Ethanol resistance was assayed on YEPD agar supplemented with 10% ethanol, and incubated at 30°C. Sc × Td clones assayed after 100 doubling are shown inside a square.

**Figure 3:**
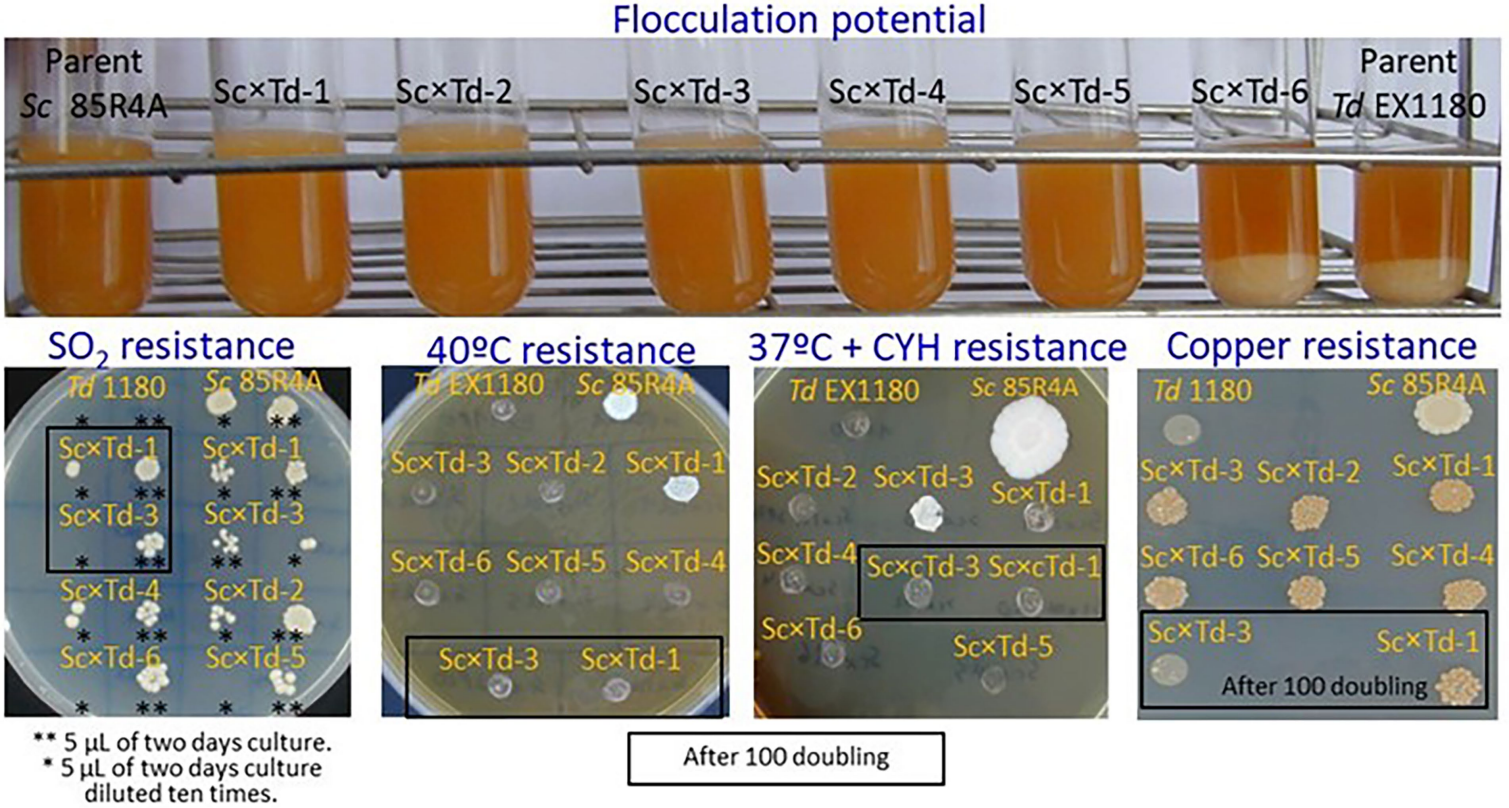
Phenotypic characterization of the *Sc*-mixed *Td* clones and their parent yeasts *Sc* 85R4A and *Td* EX1180. Flocculation potential was tested after 48 h of growth in YEPD broth at 30°C with shaking. Photographs were taken after the cultures were removed from the incubator and left for 3 minutes without shaking. Resistance to SO_2_ and Cu was tested on SD-agar plates supplemented with 125 mg/l SO_2_ and 36 mg/l Cu, respectively. Resistance to 40°C and 37°C + cycloheximide was assayed on YEPD-agar and YEPD-agar supplemented with 2 μg/ml cycloheximide, respectively.

For further biotechnological characterization, six of these *Sc*-mixed *Td* yeasts were preselected among those showing the greatest colony size on YEPD-agar. These Sc × Td yeasts showed the same phenotype than the *Td* EX1180 parent yeast for some analyzed traits such as amount of phosphate in the cell wall mannoproteins (greater in *Sc* than in *Td*), killer phenotype, and H_2_S production. Considering all of them, they only resemble the parent strain *Sc* 85R4A in the CYH^R^ phenotype (Figure 2). However, some *Sc*-mixed *Td* yeasts showed intermediate phenotype between both parent yeasts for some relevant biotechnological properties, such as flocculation potential for Sc × Td-1 to Sc × Td-5 (Figure 3), as well as resistance to ethanol (for Sc × Td-1, Sc × Td-5, and Sc × Td-6), resistance to SO_2_ (for Sc × Td-1 to Sc × Td-6), to 40°C (for Sc × Td-1), to 37°C in the presence of cycloheximide (for Sc × Td-3), and to copper (for Sc × Td-1 to Sc × Td-6). Some of these properties remained after 100 doubling for some *Sc*-mixed *Td* clones (as ethanol resistance, SO_2_ resistance, CYH resistance, and copper resistance for Sc × Td-1); while some others disappeared or became enhanced in other cases (as enhanced ethanol resistance for Sc × Td-3 and Sc × Td-1; or disappearance of CYH resistance, and copper resistance for Sc × Td-3, and high temperature resistance for Sc × Td-1; Figures 2, 3).

### Fermentation capability of *Sc*-mixed *Td* yeasts

**Figure 4:**
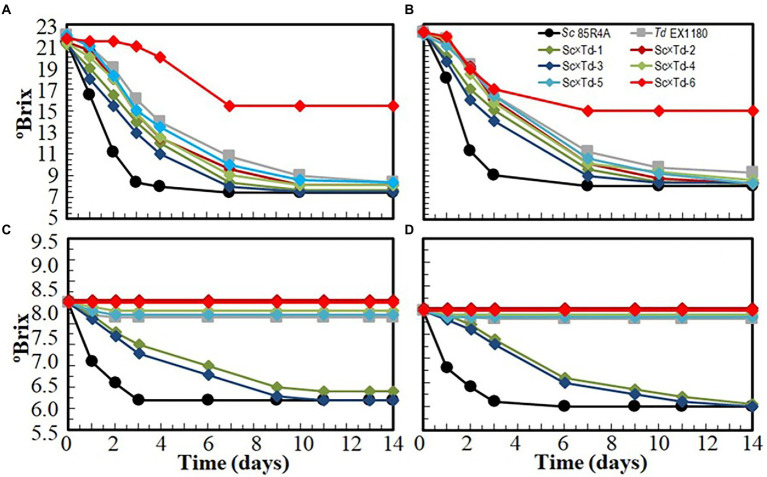
Fermentation kinetics of *Sc*-mixed *Td* clones and their parent yeasts *Sc* 85R4A and *Td* EX1180 inoculated in sterile synthetic must **(A)**, sterile synthetic must supplemented with 100 mg/l SO_2_ **(B)**, sterile synthetic base wine supplemented with 60 mg/l SO_2_ **(C)**, and sterile synthetic base wine supplemented with 100 mg/l SO_2_ **(D)**. Data are the mean values of three fermentations inoculated with each yeast strain. Standard deviations were less than 10% of the means. The degree of dominance throughout fermentation of each inoculated yeast was 100%.

The fermentation capability of these yeast mixture clones was tested in four different media, by progressively increasing the stress level of the environmental conditions. *Td* EX1180 and all *Sc*-mixed *Td* started the fermentation of sterile synthetic must later than the parent *Sc* 85R4A. However, most *Sc*-mixed *Td* yeasts showed improved fermentation kinetic as compared to the parent *Td* EX1180. The exception was Sc × Td-6, which fermentation was the slowest one, and stopped at day seven. The best *Sc*-mixed *Td* yeast was Sc × Td-3, which reached the fermentation kinetic of *Sc* 85R4A after day 7 (Figure 4A). Similar results were obtained for fermentation of synthetic must supplemented with 100 mg/l SO_2_ (Figure 4B). Only Sc × Td-1 and Sc × Td-3 completed the fermentation of the synthetic base wine supplemented with 60 mg/l SO_2_ (approximately twice as much as is usually added in commercial wineries), although they took seven to 8 days longer than the control *Sc* 85R4A. The fermentations inoculated with the rest of *Sc*-mixed *Td* yeasts and *Td* EX1180 did not start, or they stopped after 2 days (Figure 4C). Similar overall results were found for synthetic base wine supplemented with 100 mg/l SO_2_ (Figure 4D). It seems that the addition of SO_2_ to the synthetic must slightly decreased the fermentation kinetics rate of the mixed yeasts in these working conditions, but not significantly. However, the fermentation rate of synthetic base wine decreased for most yeast mixture clones as compared to that of synthetic must, although the increase in SO_2_ concentration from 60 to 100 mg/l, again, seems to not affect much. According to these results, *Sc*-mixed *Td* yeast Sc × Td-1 and Sc × Td-3 were selected for sparkling winemaking under winery conditions. At this point, it is worth noting that Sc × Td-1 was genetically more stable than Sc × Td-3. While Sc × Td-1 retained its CYH^R^ phenotype after 100 cell population doublings, most Sc × Td-3 cells lost this property (Figures 2, 3), indicating that the latter is genetically more unstable than the former. Additionally, the fermentation rate of synthetic base wine was about the same for these two Sc × Td yeasts after 100 doubling (Supplementary Figure S2). Consequently, Sc × Td-1 is presumably more suitable for industrial production and winery use than Sc × Td-3.

### Winery vinification trials and isolation of new spontaneous mutants resistant to high CO_2_ pressure (HP^R^) from *Sc*-mixed *Td* yeasts

**Figure 5:**
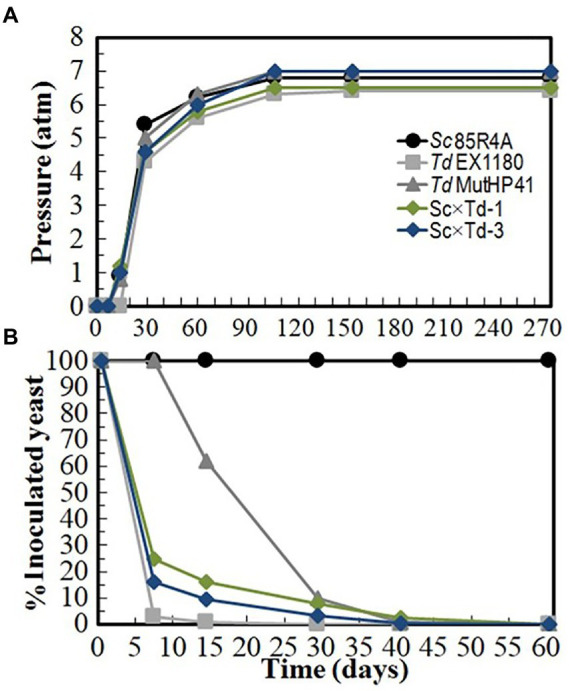
Fermentation kinetics and yeast-population dynamics of commercial base wine Macabeo I inoculated with *Sc*-mixed *Td* clones and their parent yeasts *Sc* 85R4A and *Td* EX1180. **(A)** Pressure inside the bottle. **(B)** Percentage of viable cells that belong to the inoculated yeast strain. Note that *Td* viable cells disappear as CO_2_-pressure increased. Data are the mean values of three fermentations inoculated with each yeast strain. Standard deviations were less than 15% of the means.

To assay the usefulness of the new *Sc*-mixed *Td* yeasts at industrial level, two independent sparkling wine elaboration were done with white base wine Macabeo I (at Bodegas López Morenas) and Macabeo II (at Bodegas Romale). *Td* MutHP41, which is a mutant resistant to high CO_2_ pressure (HP^R^) previously selected for sparkling winemaking (Velázquez et al., 2020), was also included in these experiments as a reference for comparison. No *Td* strain was able to dominate the process up to the end and complete the second-in-bottle fermentation of Macabeo I base wine; only the reference *Sc* 85R4A strain did it because the remaining amount of glucose + fructose was always lower than 4.1 g/l. Inoculated *Td* yeasts began to die and were overtaken by contaminant *Sc* yeasts after 20–40 fermentation days when around one to 4 atm of CO_2_ pressure was reached. In these cases, the contaminating yeasts present in the commercial base wine were responsible for completing these *Td* single-inoculated fermentations. However, if fermentation kinetics and yeast survival are considered together, Sc × Td-1 and Sc × Td-3 showed better results than the parent *Td* EX1180 strain, although not as good as *Td* MutHP41, during the first 30 days of fermentation (Figure 5). In addition, the sparkling wines inoculated with Sc × Td-1 and Sc × Td-3 showed the best foaming capability, and the best organoleptic quality score with excellent great aging notes (Supplementary Figure S3). Similar results were found for sparkling wines made with Macabeo II base wine (Supplementary Figure S4). Single-inoculated Sc × Td-3 sparkling wines contained some more reducing sugars (2.45–5.28 g/l) than Sc × Td-1 (0.76–4.89 g/l) or *Sc* 85R4A wines (0.45–4.09 g/l). These results should be carefully taken as relevant because we cannot determine how much of this result was due to the involvement of contaminant *Sc* yeasts. However, *a priori*, Sc × Td-1 again seems more suitable than Sc × Td-3 for winery applications.

**Figure 6:**
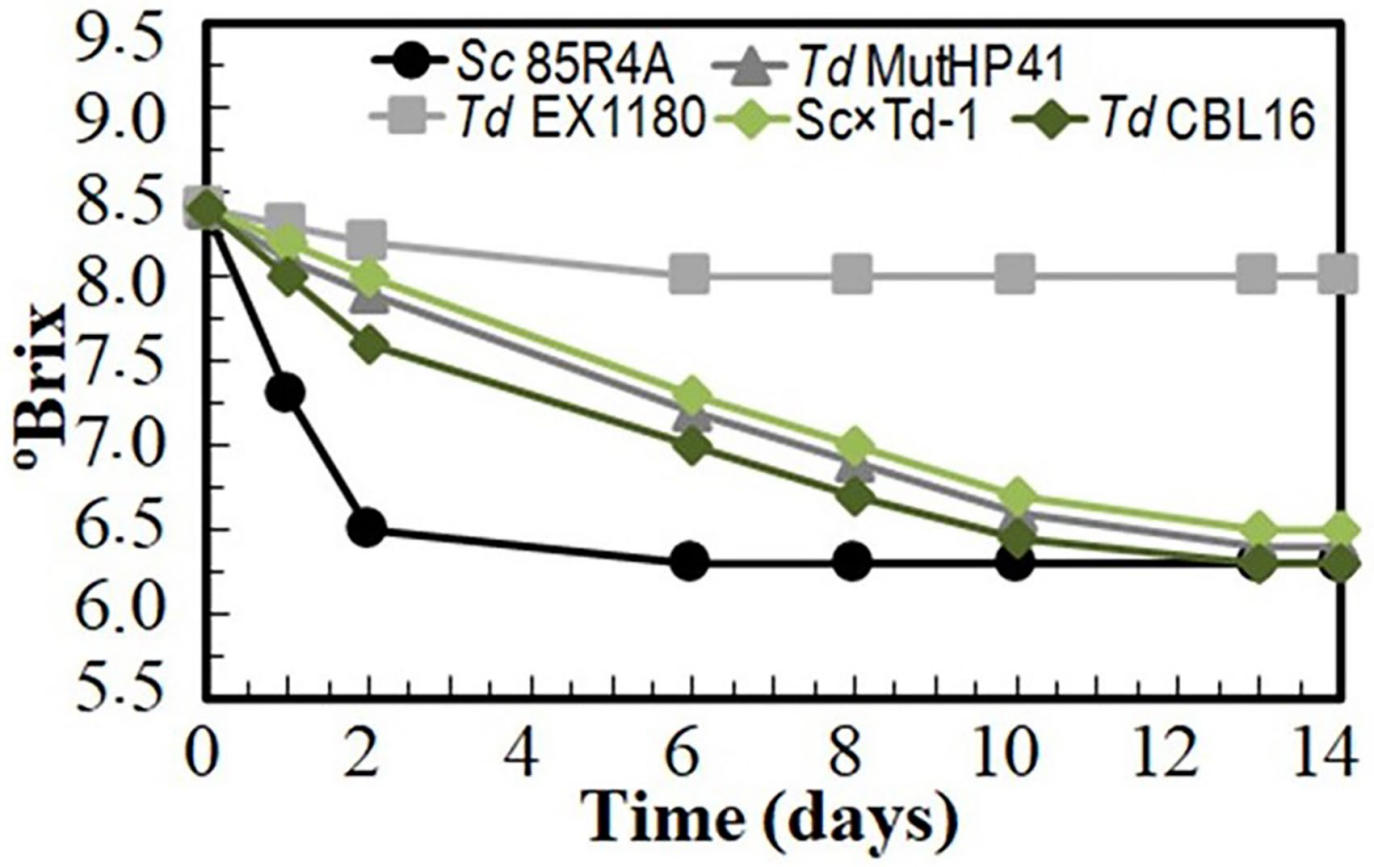
Fermentation kinetic of HP^R^ mutant *Td* CBL16 inoculated in sterile synthetic base wine supplemented with 60 mg/l as compared to its parent yeasts *Sc* 85R4A, *Td* EX1180, and Sc × Td-1, as well as to *Td* MutHP41 improved HP^R^ mutant. Data are the mean values of three fermentations inoculated with each yeast strain. Standard deviations were less than 5% of the means.

**Figure 7:**
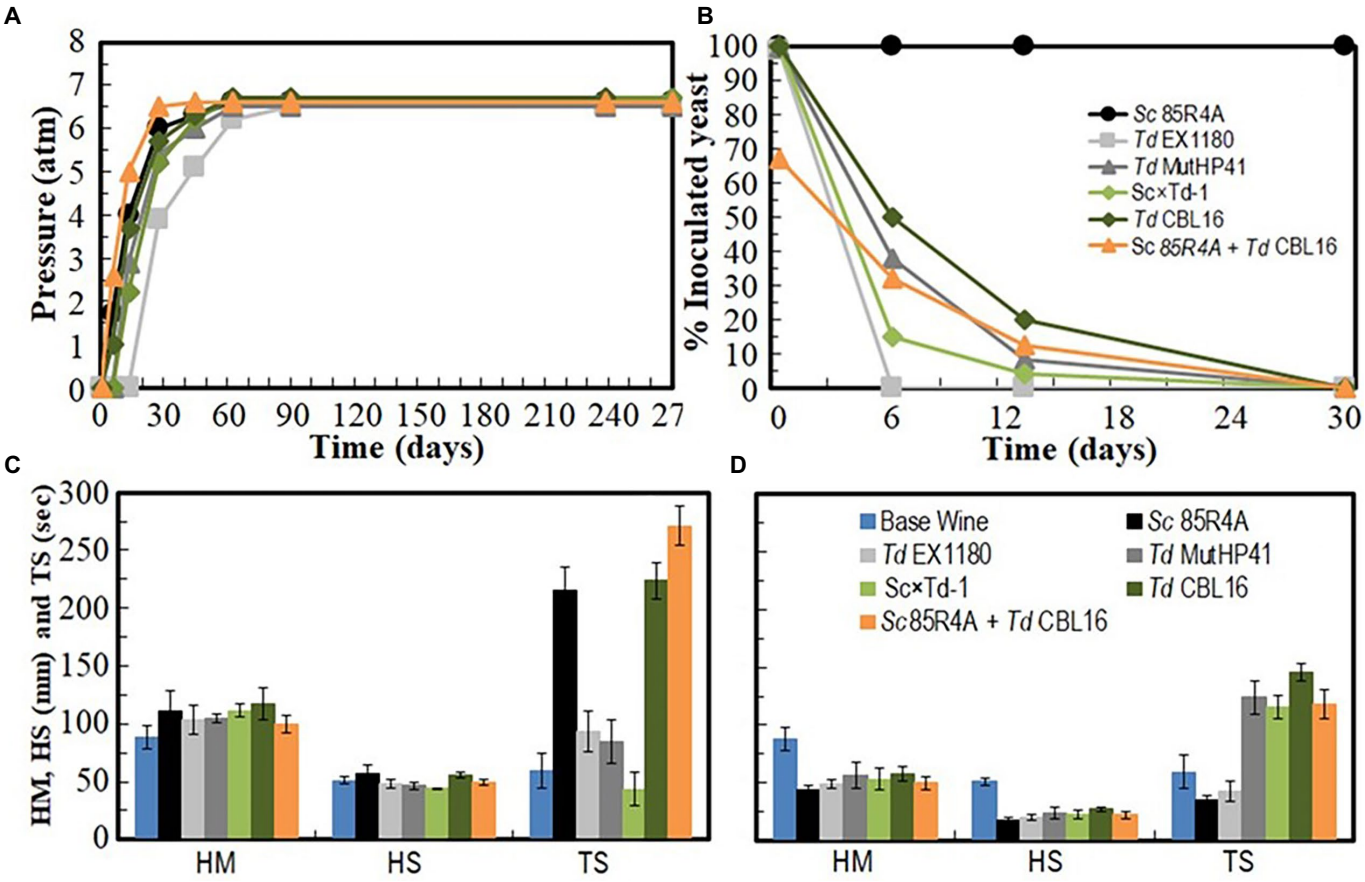
Fermentation kinetics and yeast population dynamics of commercial base wine Macabeo III inoculated with HP^R^ mutant *Td* CBL16 and its parent yeasts *Sc* 85R4A, *Td* EX1180, and Sc × Td-1, as well as with *Td* MutHP41 and a mix (1:1) of *Sc* 85R4A + *Td* CBL16. **(A)** Pressure inside the bottle. **(B)** Percentage of viable cells that belong to the inoculated yeast strain. Data are the mean values of three fermentations inoculated with each yeast strain. Standard deviations were less than 5% of the means in **(A)** and 13% of the means in **(B)**. Foaming parameters (HM, maximum height; HS, foam stability height; TS, foam stability time) after the first 30 days of fermentation **(C)** and after 9 months of fermentation and aging **(D)**.

**Figure 8:**
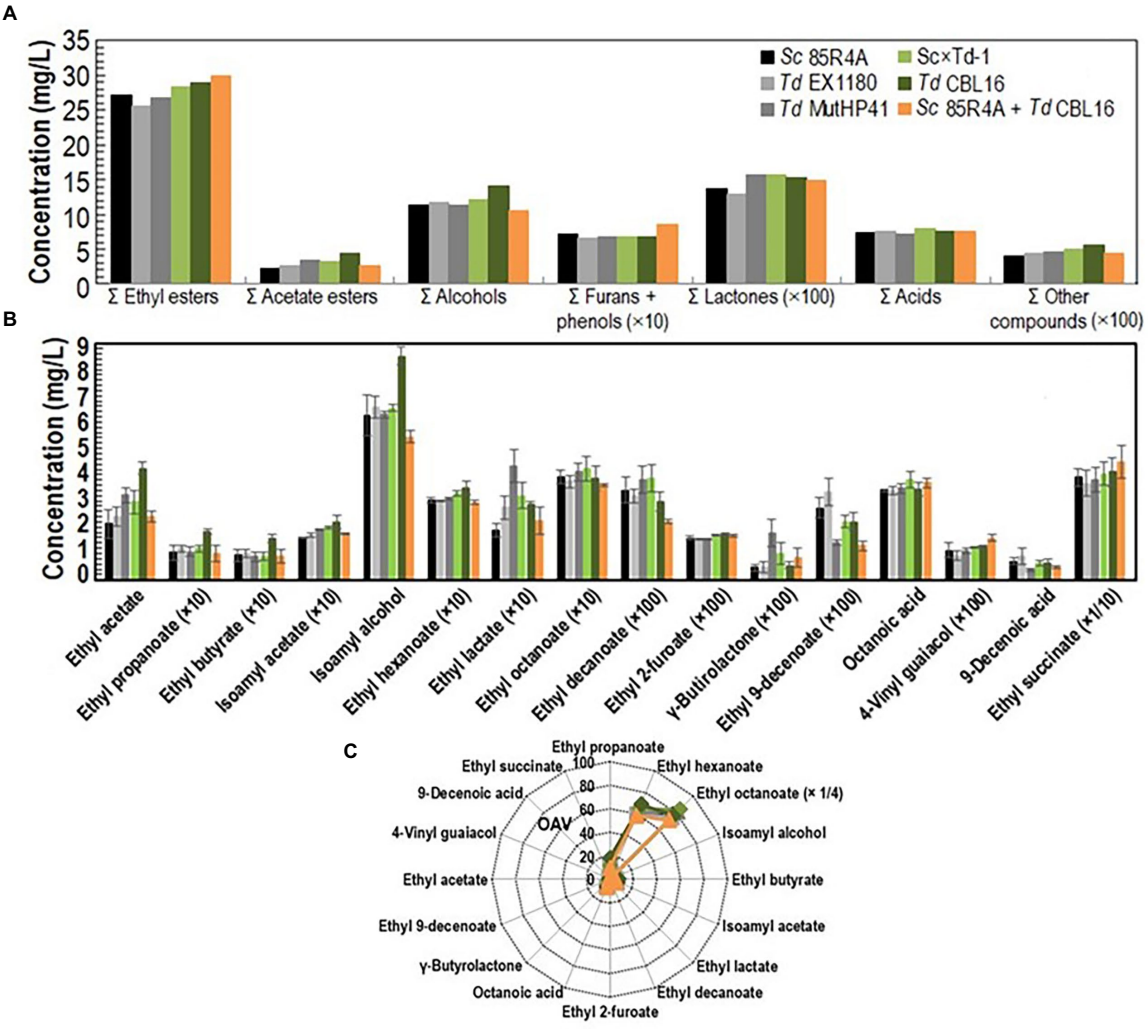
Aroma compound composition of sparkling wines elaborated with commercial base wine Macabeo III. **(A)** The amounts for the similar chemical compounds were pooled as summatory. **(B)** Aromatic compounds from which statistically significant difference (*p* < 0.05) were found between two or more wines. **(C)** Mean odor activity values (OAV) for the same aroma compounds. The three compounds with the highest OAV are shown side by side in the figure. The data are the mean ± standard error of three independent vinifications.

Several *Td* yeast colonies were isolated from YEPD plates inoculated with samples (taken at 20, 30, and 40 days of fermentation) from the sparkling wines single inoculated with *Td* yeasts. Only three colonies were isolated from sparkling wine bottles with more than 4.5 atm, and all of them came from Sc × Td-1 yeast mixture clone. The colony with the biggest size was considered a spontaneous mutant resistant to high CO_2_ pressure (HP^R^), it was named *Td* CBL16, and it was selected for further fermentation trials as it was previously done for *Td* MutHP41 (Velázquez et al., 2020). To ensure that *Td* CBL16 came from the parent *Td* yeast instead from any other contaminant yeast, its identity was verified by analyzing vegetative cell and spore morphology, killer phenotype, cycloheximide resistance, presence of viral dsRNA, mtDNA RFLPs, and sequencing of ITS genomics regions. We confirmed that *Td* CBL16 conserved the cycloheximide resistance as well as all the phenotypes that its parent yeast Sc × Td-1 showed before 100 doublings (Figures 2, 3). *Td* CBL16 showed improved fermentation kinetic of synthetic base wine supplemented with 60 mg/l as compared to its parent yeast strain Sc × Td-1, as well as compared to *Td* MutHP41 mutant (Figure 6). Therefore, *Td* CBL16 was selected for a new sparkling-wine vinification trial in our experimental winery using Macabeo III commercial base wine. Co-inoculation with *Sc* 85R4A + *Td* CBL16 (1,2) was also assayed for comparison. Once again, *Td* CBL16 improved fermentation kinetic with respect to their parent yeasts *Td* EX1180 and Sc × Td-1, and now also with respect to *Td* MutHP41. The fastest fermentation was the one co-inoculated with *Sc* 85R4A + *Td* CBL16, probably because these bottles contained more yeast cells than the rest at the starting point. After 10 days of fermentation, *Td* CBL16-inoculated sparkling wine reached almost the same CO_2_ pressure than *Sc* 85R4A-inoculated wine, 3.7 ± 0.15 vs. 4.0 ± 0.1 atm, respectively (Figure 7A). *Td* CBL16 cells also remained viable in larger proportion than the rest of *Td* yeasts after the first 30 days of fermentation, even when co-inoculated with *Sc* 85R4A (Figure 7B). After 30 days of second-in-bottle fermentation, the foaming capability of *Td* CBL16 wines was slightly better than that of the rest of *Td* yeast inoculated wines, and close to that of *Sc* 85R4A inoculated wine (Figure 7C). However, the foaming capability of all sparkling wines (mainly HM and HS) decreased and became similar after 9 months of fermentation and aging. The exception was the TS value for *Td* CBL16, Sc × Td-1, *Td* MutHP41, and *Sc* 85R4A + *Td* CBL16 wines, which remained or increased to become the best ones (Figure 7D). This overall decreasing of foaming capability could be because of the participation of *Sc* yeasts to complete the second-in-bottle fermentation. Although all wines showed similar enological parameters and good organoleptic quality, Sc × Td-1, *Td* CBL16, and *Sc* 85R4A + *Td* CBL16 wines were the most appreciated by the experts (Table 1), mainly because they showed pleasant fruity and excellent aging notes at the same time. This may be because of the increased participation of these Sc × Td-1 and *Td* CBL16 yeasts during the sparkling wine fermentation as compared to their parent *Td* EX1180 (Figure 7B), and to the greater amount of ethyl and acetate esters in their wines with respect to the wines of their parent yeast *Td* EX1180. The amount of ethyl esters was similar in Sc × Td-1, *Td* CBL16 and *Sc* 85R4A wines (Figure 8A). Possible relevant differences were observed for 16 out of the 75 volatile compounds analyzed (Figure 8B). Among these compounds, the greatest odor activity values (OAVs) corresponded to three compounds with fresh fruit odor descriptors: ethyl propanoate (banana, apple), ethyl hexanoate (banana, green apple), and ethyl octanoate (banana, pineapple, pear, floral); although other compounds such as ethyl butyrate (grape, apple skin) and isoamyl acetate (banana, fruit) may also contribute to the aroma of these wines (Figure 8C). The increased amount of ethyl propanoate, ethyl hexanoate, ethyl butyrate, and isoamyl acetate in *Td* CBL16, *Td* Sc × Td-1, and *Td* MutHP41 wines with respect to the parent *Td* EX1180 may explain the increased organoleptic quality rate of these wines, as well as for the *Sc* 85R4A + *Td* EX1180 mixed inoculated wine.

**Table 1:** Some relevant parameters and organoleptic quality of sparkling wines (cavas) made from commercial base wine Macabeo III single or mixed inoculated with different yeasts.

Inoculated yeast	Alcohol (%, *v*/*v*)	pH	Total acidity(g/l)	Volatile acidity(g/l)	Glucose + fructose (g/l)	Malic acid (g/l)	Lactic acid (g/l)	Citric acid (g/l)	Preference (%)
*Sc* 85R4A	10.76	3.06	5.14	0.11	0.15	0.53	0.08	0.25	83
*Td* EX1180	10.73	3.04	4.73	0.16	0.88	0.01	0.41	0.05	68
*Td* MutHP41	10.70	3.06	4.95	0.14	0.78	0.03	0.41	0.10	85
*Td* Sc × Td-1	10.75	3.04	4.99	0.14	0.60	0.03	0.40	0.12	86
*Td* CBL16	10.70	3.06	4.99	0.11	0.45	0.09	0.34	0.21	92
*Sc* 85R4A + *Td* CBL16	10.72	3.06	5.18	0.14	0.12	0.08	0.33	0.16	88

The data are the means of two independent measurements after 9 months of wine aging and disgorging. The standard deviation was always less than 9% of the mean.

Given that *Td* yeasts have greater oxygen requirement to grow than *Sc* yeasts, conditioning of yeast culture with occasional or continuous shaking before inoculation of synthetic base wine was tested. The continuous-shaking culture adaptation, which supplies extra amount of oxygen, was the most effective procedure to improve the fermentation capability of all tested yeasts, especially that of *Td* yeasts. Interestingly, *Td* Sc × Td-1, *Td* CBL16, and *Td* MutHP41 showed the better improvement, with °Brix values like those of the parent *Sc* 85R4A after 7 fermentation days (Supplementary Figure S5).

## Discussion

### Characterization of the new *Sc*-mixed *Td* yeasts

Yeast mixture clones were obtained by mixing vegetative cells of diploid *Sc* 85R4A and haploid *Td* EX1180. Cell and spore morphology, sequence of the ribosomal RNA gene spacers (ITS), and flow cytometry analysis indicated that these yeast mixture clones are *Td* yeasts as the parent *Td* EX1180 yeast. They probably were Kbarr-1 CYH^R^ yeasts originated from any type of rare mating between *Sc* and *Td* vegetative cells, which thereafter became segregant haploid yeasts raised from these hybrids that should be genetically unstable. Alternatively, this Kbarr-1 CYH^R^ yeasts may have arisen by some type of lateral gene transfer from *Sc* to *Td*. However, this seems to be unlikely because we found the transfer of several *Sc* properties to the same *Sc*-mixed *Td* clone, which may require the transfer of several genes or even part of several chromosomes from *Sc* to *Td*. Another possibility is the transfer of some regulatory RNA molecule from *Sc* to *Td* by some mechanism as extracellular vesicles (Mencher et al., 2020). Further research should be accomplished to clarify this issue.

No mixture yeast clone was obtained from mixtures of sporulated cultures of the same parent yeasts. This probably was because there was no mating between germinated spores from the two parent strains, and thereafter all the *Sc* cells died in 4 MB plates because of the presence of active Kbarr-1 toxin from *Td* cells.

In any case, what is important to improve *Td* fermentation efficiency is that transfer of some phenotypic characteristics from *Sc* to *Td* was found in the new mixture of yeast clones. Some of these characteristics are interesting technological traits for sparkling winemaking, such as low flocculation capacity to avoid sticky or voluminous sediments of yeasts, or SO_2_ and ethanol resistances that should allow the yeasts to remain viable longer during the fermentation of base wine, which usually contains SO_2_ and above 9% ethanol. Interestingly, most of the selected *Sc*-mixed *Td* yeasts improved the fermentative capability of synthetic must with respect to their *Td* parent strain. And two of them, Sc × Td-1 and Sc × Td-3, were also able to complete the fermentation of synthetic base wine in the presence of SO_2_ and the absence of CO_2_ pressure, showing fermentation kinetics close to that of their parent *Sc* strain. Moreover, for these two yeast mixture clones, it appears that increasing the SO_2_ concentration from 60 to 100 mg/l only slightly decreased the fermentation rate. For all these reasons, we considered it interesting to select the *Sc*-mixed *Td* yeast Sc × Td-1 and Sc × Td-3 for winery trials.

No changes were observed in the amount of phosphate present in the cell wall mannoproteins of the yeast mixture clones with respect to the *Td* parent yeast. However, the flocculation capacity of most mixture clones was reduced, as compared to the *Td* parent yeast, reaching a low level of flocculation like that of the *Sc* parent. This indicates that the absence of positive charge on the surface of the *Td* yeasts due to the absence of phosphate in cell wall mannoproteins should not be a determining factor for these yeast cells to aggregate to form flocs. It has been suggested that yeast flocculation can be mainly due to protein lectins on the yeast cell surface that interact with either the mannose-containing and/or the glucose-containing carbohydrates present on the cell wall surface of adjacent cells (Stewart, 2018). Assuming this proposal to explain our results, it is possible that the parent *Sc* transferred some genetic information to the parent *Td* that suppressed the synthesis of protein lectins located on the cell surface of the *Sc*-mixed *Td* yeasts that became non-flocculent.

### Fermentation capability and winery use of the selected *Sc*-mixed *Td* yeasts

For industrial sparkling winemaking, with increasing CO_2_ pressure inside of a bottle, the fermentation kinetics and cell viability of the new *Sc*-mixed *Td* yeasts were better than those of the parent *Td* yeast. However, these results were not as good as those previously obtained for *Td* MutHP41 (Velázquez et al., 2020). Consequently, isolation of new HP^R^ mutants from the *Sc*-mixed *Td* yeasts was required. Fortunately, one among these HP^R^ mutants, *Td* CBL16, showed better fermentation kinetics and cell survival than *Td* MutHP41. Additionally, the organoleptic and foam quality of *Td* CBL16-inoculated wines was the best compared to all *Td*-inoculated sparkling wines. Moreover, it surpassed the quality of the wine made with the reference *Sc* yeast. This result suggests that single inoculation with *Td* CBL16 yeast a suitable strategy for producing sparkling wine of reliable quality, ensuring the maximum possible participation of *Td* yeasts in this technological process. Moreover, the continuous oxygen supply during the previous adaptation of the new *Sc*-mixed *Td* yeasts to the base wine environment further improved their resistance to ethanol and, therefore, their fermentation capability, as it was previously found for *Td* MutHP41 (Velázquez et al., 2020). Beside this, the genetic stability of *Td* Sc × Td-1 and its new HP^R^ mutant *Td* CBL16 after 100 doublings in rich non-selective culture medium (Ramírez et al., 2004) indicates that these *Sc*-mixed *Td* yeasts are stable enough to be produced at industrial scale and marketed for sparkling winemaking, without the risk that they may lose their biotechnological properties.

Since fermentation capability of these *Sc*-mixed *Td* yeasts is still lower than that of *Sc*, co-inoculation with this latter yeast may still be required to ensure that second fermentation of commercial sparkling wine is completed in a reasonable time. This could be especially concerning if commercial base wine lacks appropriate contaminant *Sc* yeasts to complete second fermentation, preferably within 2 months. The novelty of using these new *Sc*-mixed *Td* yeasts is that they would remain viable longer than their *Td* parent yeast during second fermentation of base wine, even when these yeasts are co-inoculated with *Sc* yeasts. This should enhance the organoleptic characteristics coming from *Td* yeast in sparkling wine.

We are currently continuing to test these new yeasts in wineries to validate their ability to improve the quality of sparkling wine or, alternatively, to make differentiated wines. At the same time, we are obtaining new parent yeasts with several new genetic markers, which hopefully would allow us to further analyze the nature of the transfer of genetic information from *Sc* to *Td* yeasts through the mixture for eventual hybridization technique.

## Conclusion

Transfer of some interesting technological characteristics from *Sc* to *Td* was obtained by mixing yeast vegetative cells. This allowed *Td* yeasts to remain viable longer during sparkling wine second fermentation. The new *Sc*-mixed *Td* yeasts showed improved fermentation kinetics and cell survival than a previously selected *Td* HP^R^ mutant. The organoleptic and foam quality of *Td* CBL16-inoculated wines was improved, even above the sparkling wine made with the reference *Sc* yeast. Additionally, yeast mixture clones are stable enough to be marketed for industrial sparkling winemaking. This success encourages more research to find out whether the transfer of other technological traits from *Sc* to *Td*, or to other non-conventional yeasts, can occur.

## Data availability statement

The raw data supporting the conclusions of this article will be made available by the authors, without undue reservation.

## Author contributions

MR conceived the project and wrote and edited the manuscript. MR, AM, EZ, and MÁ designed and performed the experiments. MR, AM, and JB-G analyzed the data. All authors contributed to the article and approved the submitted version.

## Funding

This work was funded by grants GR21062, IB16132, and IB20069 from Extremadura Regional Government (Consejería de Economía, Ciencia y Agenda Digital); and AGL2017-87635-R from Spanish Ministry of Education and Science, and European Regional Development Fund (ERDF-European Union).

## Conflict of interest

The authors declare that the research was conducted in the absence of any commercial or financial relationships that could be construed as a potential conflict of interest.

## Publisher’s note

All claims expressed in this article are solely those of the authors and do not necessarily represent those of their affiliated organizations, or those of the publisher, the editors and the reviewers. Any product that may be evaluated in this article, or claim that may be made by its manufacturer, is not guaranteed or endorsed by the publisher.

## Footnotes

^1^ http://ncbi.nlm.nih.gov

^2^ http://ncbi.nlm.nih.gov
