# Supplementary material for: Genetic improvement of non-conventional Torulaspora delbrueckii for traditional sparkling winemaking by mixing for eventual hybridization with Saccharomyces cerevisiae
Source: Front Microbiol. 2022 Oct 6;13:1006978. doi: 10.3389/fmicb.2022.1006978 (PMC9583163; doi:10.3389/fmicb.2022.1006978)
Supplement: Supplementary file 1 [file Data_Sheet_1.pdf]

Supplementary Material

Supplementary Figures

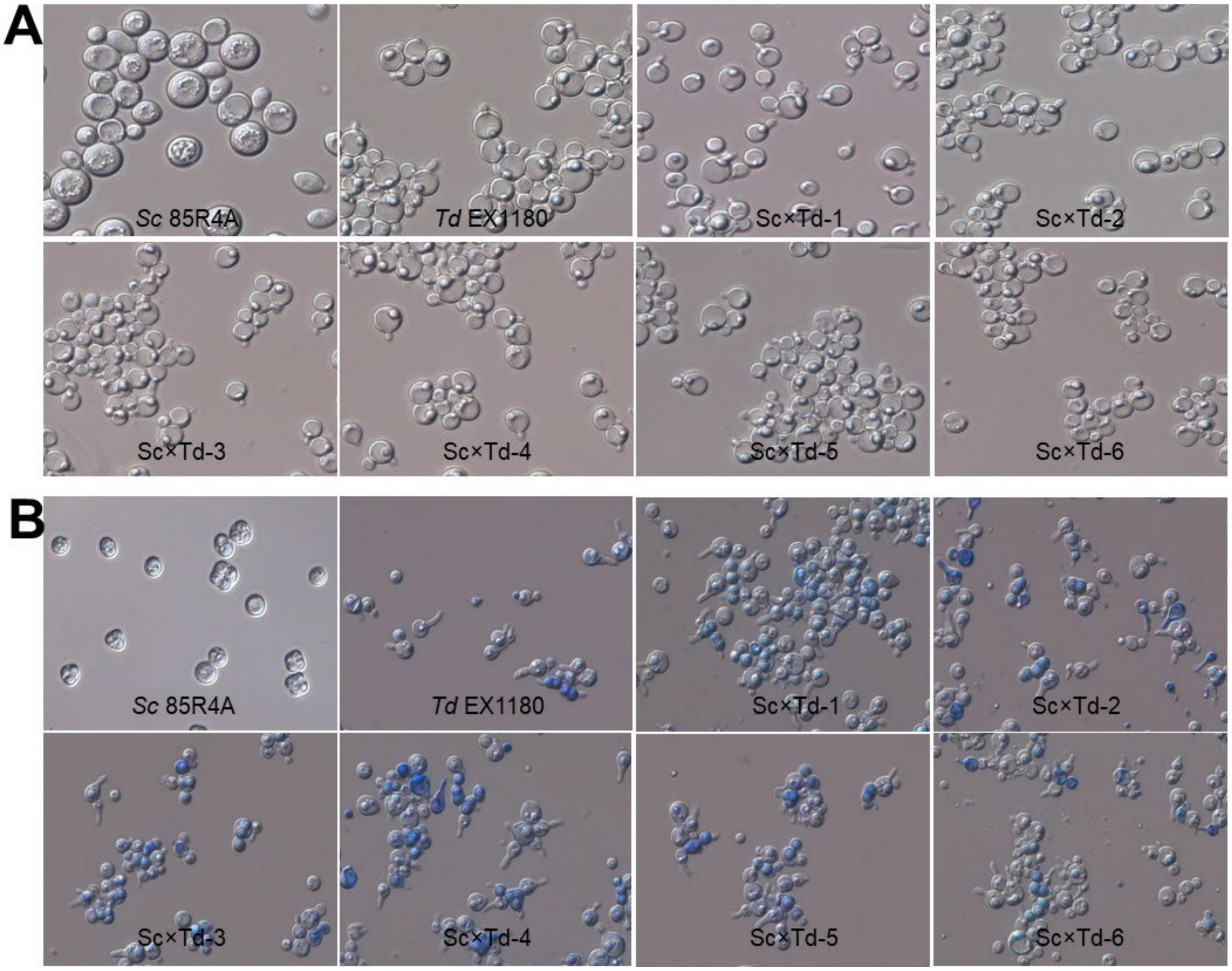

Supplementary Figure S1. Vegetative cells **(A)** and spores **(B)** of *Sc* 85R4A and *Td* EX1180 parent yeasts, and six Sc-mixed *Td* clones (Sc×Td-1 to Sc×Td-6) for comparison. These photomicrographs were taken with a Nikon Eclipse 600 microscope, equipped with a 60× objective, using Nomarski interference contrast.

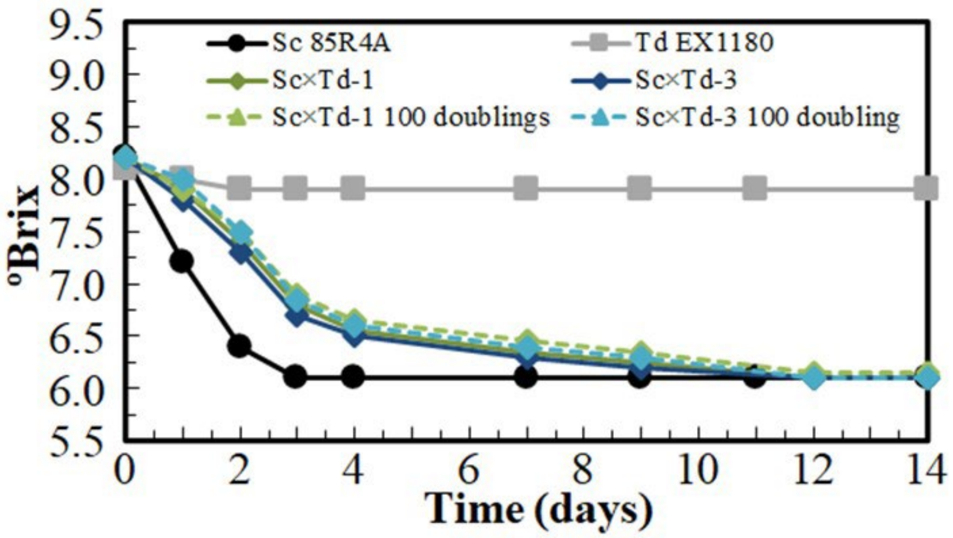

Supplementary Figure S2. Genetic stability of Sc×Td-1 and Sc×Td-3 yeasts after 100 doublings of cell population. Fermentation kinetics of sterile synthetic base wine (60 mg/L SO_2) inoculated with yeast cultures conditioned with occasional shaking. Data are the mean values of three fermentations inoculated with each yeast strain. Standard deviations were less than 5% of the means. The degree of dominance throughout fermentation of each inoculated yeast strain was 100%.

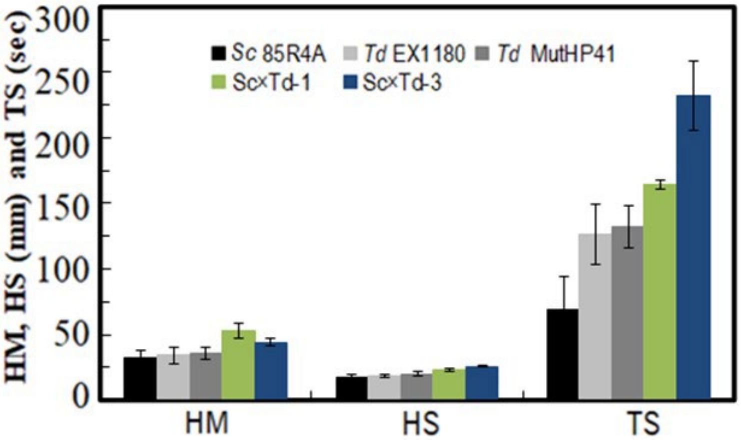

Inoculated yeast	Alcohol (%, v/v)	pH	Total acidity (g/L)	Volatile acidity (g/L)	Glucose + fructose (g/L)	Malic acid (g/L)	Lactic acid (g/L)	Citric acid (g/L)	Preference (%)
Sc 85R4A	11.9	3.23	4.3	0.29	4.09	0.04	0.70	0.02	80
Td EX1180	12.3	3.17	4.5	0.25	5.92	0.07	0.66	0.05	72
Td MutHP41	12.1	3.26	4.6	0.28	5.27	0.06	0.66	0.03	81
Sc×Td-1	11.7	3.31	4.4	0.26	4.89	0.05	0.68	0.02	87
Sc×Td-3	11.7	3.24	4.5	0.25	5.28	0.09	0.67	0.02	85

The data are the means of two independent measurements after nine months of wine aging and disgorging.
The standard deviation was always less than 7% of the mean.

Supplementary Figure S3. Foaming parameters (HM, maximum height; HS, foam stability height; TS, foam stability time) and some relevant parameters of Macabeo I sparkling wines after nine months of fermentation and aging.

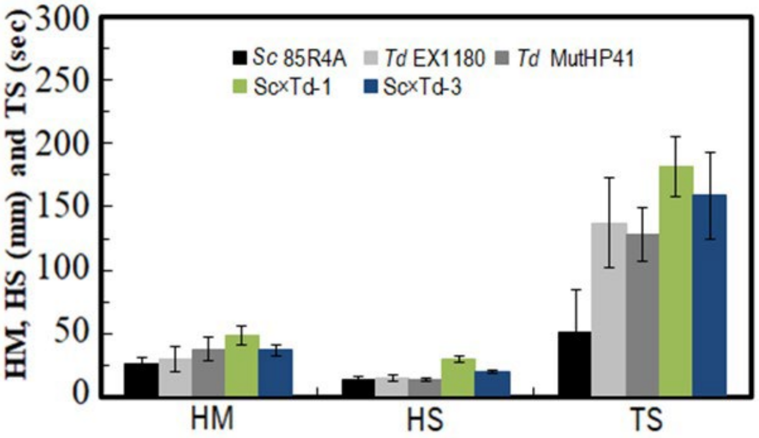

Inoculated yeast	Alcohol (%, v/v)	pH	Total acidity (g/L)	Volatile acidity (g/L)	Glucose + fructose (g/L)	Malic acid (g/L)	Lactic acid (g/L)	Citric acid (g/L)	Preference (%)
Sc 85R4A	11.5	2.93	5.7	0.19	0.45	1.09	0.07	0.22	84
Td EX1180	11.3	2.86	4.3	0.17	0.66	1.10	0.07	0.24	75
Td MutHP41	11.5	2.84	5.0	0.16	0.86	1.09	0.07	0.24	85
Sc×Td-1	11.5	2.82	5.2	0.17	0.76	1.11	0.07	0.25	88
Sc×Td-3	11.2	2.83	5.4	0.15	2.45	1.12	0.06	0.24	90

The data are the means of two independent measurements after nine months of wine aging and disgorging.
The standard deviation was always less than 9% of the mean.

Supplementary Figure S4. Foaming parameters (HM, maximum height; HS, foam stability height; TS, foam stability time) and some relevant parameters of Macabeo II sparkling wines after nine months of fermentation and aging.

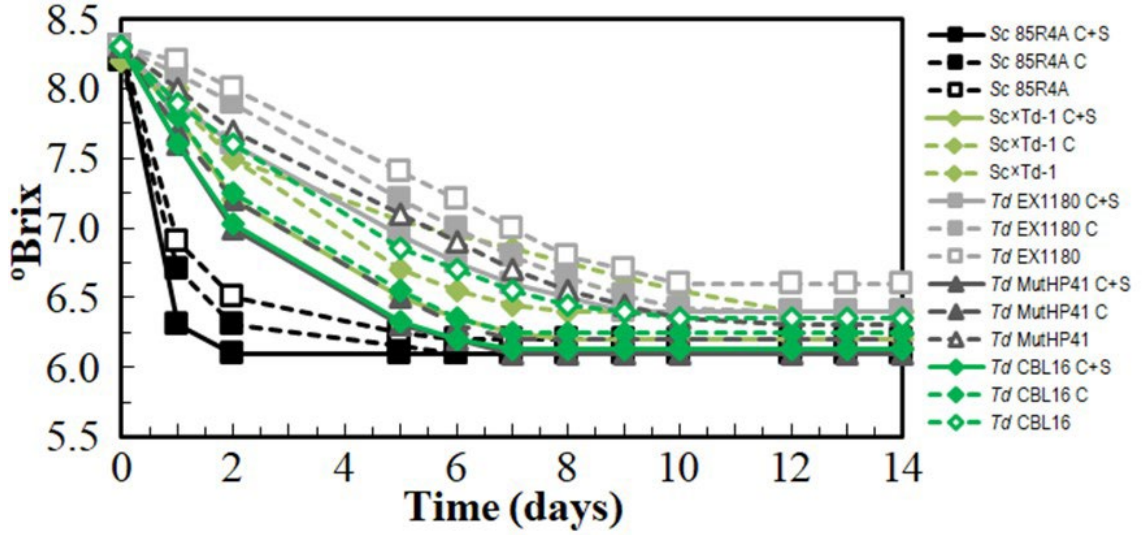

Supplementary Figure S5. Fermentation kinetics of sterile synthetic base wine (30 mg/L SO_2) inoculated with *Td* CBL16 and its parent yeasts *Sc* 85R4A, *Td* EX1180, and Sc×Td-1, as well as with *Td* MutHP41. Before inoculation, the yeast cultures were unconditioned (no indication following the name of the strain), conditioned with occasional shaking (C), or conditioned with continuous shaking (C+S). Data are the mean values of three fermentations inoculated with each yeast strain. Standard deviations were less than 6% of the means. The degree of dominance throughout fermentation of each inoculated yeast strain was 100%.
